# Physio-biochemical analysis and molecular characterization of induced lentil mutant lines

**DOI:** 10.1371/journal.pone.0274937

**Published:** 2022-10-24

**Authors:** Durre Shahwar, Mohammad Yunus Khalil Ansari, Younghoon Park

**Affiliations:** 1 Cytogenetics and Molecular Biology laboratory, Department of Botany, Aligarh Muslim University, Aligarh, India; 2 Department of Horticultural Bioscience, Pusan National University, Miryang, Korea; Huazhong University of Science and Technology, CHINA

## Abstract

*Lens culinaris* is a proteinaceous food crop that is consumed worldwide for protein requirements. Mutation breeding has been used to improve protein content, yield, and related traits, as well as to select highly desirable mutants that are economically significant. An investigation of genotypic variation in lentil germplasm was carried out using induced mutagenesis, with caffeine, ethyl methane sulfonate (EMS), lead nitrate, and cadmium nitrate as mutagens that resulted in 18 mutant lines in the M_3_ generation. For the present study, we analyzed the genetic diversity of lentil mutant lines using sodium dodecyl sulfate-polyacrylamide gel electrophoresis and random amplified polymorphic DNA markers (RAPD). The heterozygosity of RAPD markers per primer ranged from 50.00–90.90% with an average of 71.04%. The genetic divergent analysis was performed using hierarchical clustering (UPGMA), exhibited that these mutant lines were classified mainly into five subpopulation or clusters. A close resemblance with highest genetic coefficient similarity (1.00) were observed between control and mutant H; between mutant M and E; between mutant Q and J_2,_ while more divergent mutants were N_2_ with mutant B; and mutant R with mutant J_1_with least genetic coefficient similarity (0.22). Protein and mineral content (Fe, Zn and Cu) were increased significantly in some high yielding mutant lines concerning to the control plant, and showed polymorphic variations in polypeptide chains in terms of banding pattern. Stomatal morphology in high yielding mutants were perceived utilizing scanning electron microscopy (SEM), exhibiting variations in stomatal size, stomatal opening and number of stomata. The present study’s promising mutant lines’ biological, physiological, and molecular profiles provide a foundation for forthcoming preservation and consumption strategies to broaden the genetic diversity of the breeding population of lentil.

## Introduction

Legumes are known to be crucial agricultural food crops enhancing the nutrition and income of farmers comprehensively across the world. It also plays a pivotal role in providing food and nutritional security. In India, the primarily grown pulses are chickpea, lentil, mungbean, pigeon pea, and urdbean. According to the FAO [1], the total area for lentil production is 16,575/kilometers [2] having a production of about 12,20,000 tons at a rate of 7,36 kilogram/ hectare yield. Lentil production in India ranks second globally with an annual production of roughly 6.3 million tons of the total world production. Lentil is an annually grown, self-pollinating diploid plant with a haploid genome size of 4063Mbp [3] containing high protein, fibers, many essential vitamins and minerals. Besides, it is also an accomplished source of complex carbohydrates, vegetable proteins, and low amount of fat free cholesterol [3]. The lentil yield was low compared to the world average [1] indicating a lack of efficient agricultural practices. However, after massive research efforts, the low yield potential of lentil remains a major restriction for obtaining lentil production goals. Consequently, in order to overcome the issue of limited genetic variability and production of superior cultivars with high yield along with other quantitative traits, a systematic breeding method is required.

Among conventional and modern methods of plant breeding, mutation breeding is one of the most rapid, worthwhile, and effective approach to improve genotypes with improved agronomic traits by altering genotypes. A variety of morphological, physiological, biochemical, and molecular markers are utilized to assess genetic variability accurately and quickly. Since environmental factors and plant developmental stages have a major effect on the morphological characteristics of the plant, Lee [4] and Srivastava et al. [5] pointed out that the evaluation of genetic diversity based on phenotypic characters of plants that has been used for a long time in plant breeding, has certain limitations. With the advancement in genomic techniques and molecular markers, these agronomic traits/characters are now amplified. The evaluation of genetic variability in the seed storage protein in mutagenized population of lentil using electrophoresis technique is very meager. The researchers can now evaluate genetic variation due to the advent of molecular techniques. The atomic absorption spectrometry is also helpful in determining mineral content for isolating and screening new mutants/cultivars and establishing genetic diversity in mutagenized plants. The effects of mutagens on trace elements in cells and their genetic variation have not been studied very thoroughly. Therefore, the work on the enhancement of mineral elements in pulses needs to be undertaken.

The study was planned to identify the possible mutant lines and characterize the mutant’s genotype after continuous selection from M_1_ to M_3_ generations followed by biochemical (seed protein and mineral nutrients), physiological (stomatal morphology) and molecular (SDS PAGE and RAPD) based analysis (Table 1).

**Table 1:** List of selected mutants of *Lens culinaris* Medik. in M_3_ generation for molecular analysis.

S. No.	Control/mutant code	Mutagens used	Concentrations (%/ppm)	Remarks
1	CN	-	-	Normal yield
2	A	Caffeine	0.10	Tall mutant with increased yield
3	B	Caffeine	0.25	Semi-dwarf mutant with slightly improved yield
4	C	Caffeine	0.50	Tall mutant with high yield
5	D	Caffeine	0.75	Dwarf mutant with low yield
6	E	Caffeine	1.0	Dwarf mutant with low yield
7	G	EMS	0.10	Tall mutant with high yield
8	H	EMS	0.25	Tall mutant with increased yield
9	I	EMS	0.50	Mutant with average height and moderately increased yield
10	J_1_	EMS	0.75	Semi-dwarf mutant with increased yield
11	J_2_	EMS	0.75	Semi-dwarf mutant with an average yield
12	L	Pb(NO_3_)_2_	20	Bushy mutant with average height, slightly improved yield
13	M	Pb(NO_3_)_2_	40	Semi-dwarf mutant with moderately increased yield
14	N_1_	Pb(NO_3_)_2_	60	Dwarf mutant with moderate yield
15	N_2_	Pb(NO_3_)_2_	80	Dwarf mutant with low yield
16	P	Cd(NO_3_)_2_	20	Dwarf mutant with moderately increased yield
17	Q	Cd(NO_3_)_2_	60	Semi-dwarf mutant with low yield
18	R	Cd(NO_3_)_2_	80	Dwarf mutant with low yield
19	S	Cd(NO_3_)_2_	100	Semi dwarf mutant (sterile)

## Materials and methods

### Plant materials

Eighteen lentil mutants were used in this study. These mutants were selected from M_1_ to M_3_ generation continuously based on morphological characters. The Indian Agricultural Research Institute in New Delhi, India provided the parental genotype (*Lens culinaris* Medik. var. L-4076).

### Experimental procedure of mutant lines selection

**Fig 1:**
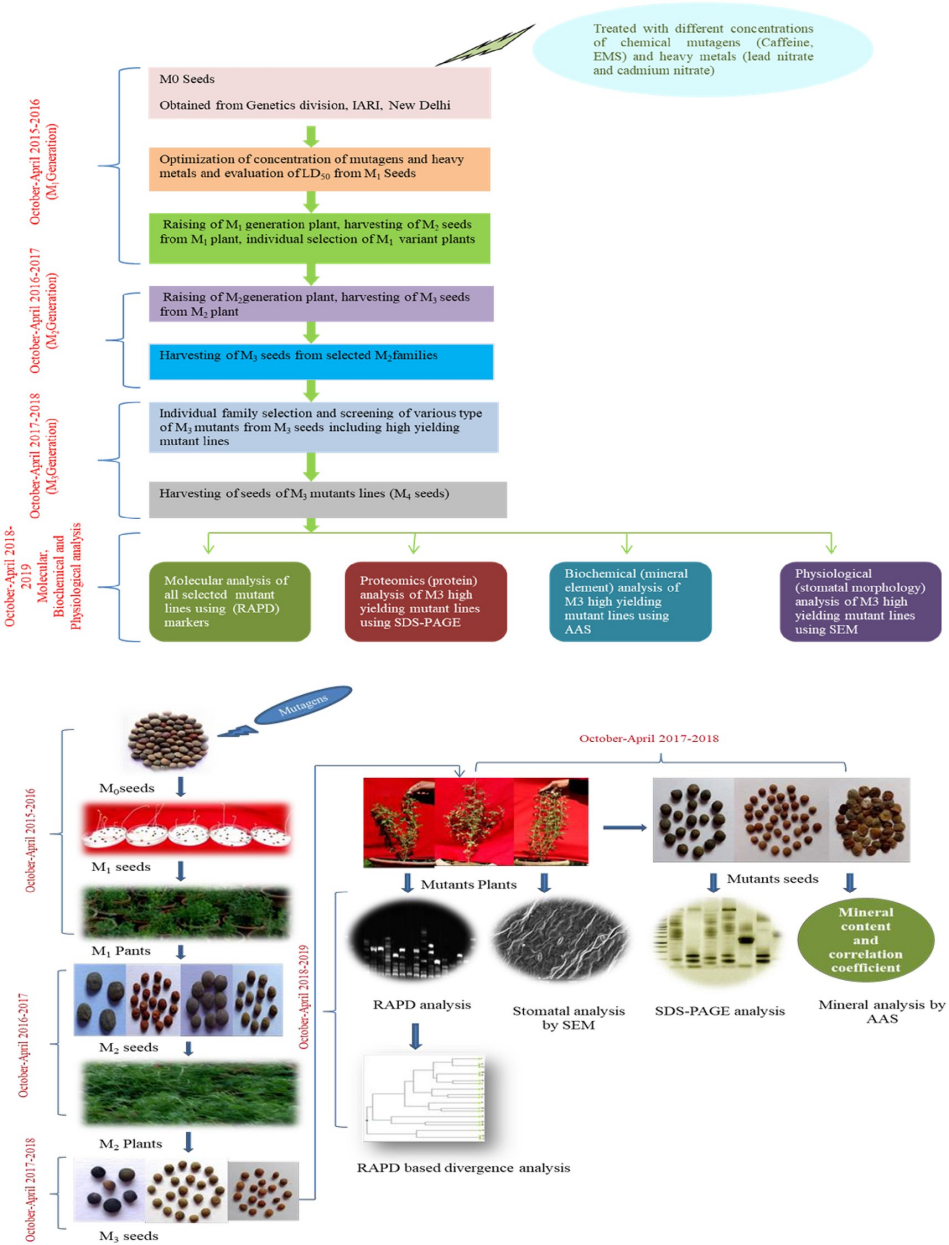
Figure of chemical mutagenesis and mutant selection procedure during 2015–2019.

The mutation breeding program is presented in (Fig 1). Different concentrations (v/v) of caffeine and EMS 0.10%, 0.25%, 0.50%, 0.75%, and 1.0%, were applied for 12 hours to the dried and certified seeds after a pre-soaking in water at room temperature. Also, different sets of seeds were prepared by treating heavy metals (lead nitrate and cadmium nitrate) at concentrations like 20ppm, 40ppm, 60ppm, 80ppm and 100ppm. The mutagenic treatment of heavy metals and chemical mutagens are described in detail in previous study related to the work [6, 7]. Initially, LD50 values were first calculated using germination rate to optimize the mutagenic treatments. One hundred seeds from each treatment and control populations were grown in 5 replicates (20 seeds in one pot) to raise M_1_ generation. All the seeds were harvested from M_1_ plants separately and were sown treatment-wise in pots for raising M_2_ generation. From M_2_ generation onwards, at each subsequent generation 20 viable, significant mutant lines were selected from each 0.10%, 0.25%, 0.50%, 0.75% and 1.0% concentrations of caffeine and EMS, and 20ppm, 40ppm, 60ppm, 80ppm and 100ppm lead and cadmium nitrate treated population based on morphological characters and yield. Therefore, a total of 400 M_2_ mutant lines were selected for raising subsequent M_3_ generation. Further, for the purpose of M3 generation propagation, 10 M3 seeds from each 400 M_2_ mutant lines were taken into consideration. In M_3_ generation, 10 morphological and 8 high yielding mutant lines were screened and selected from different treatments of caffeine, EMS, Pb(NO3)_2_ and Cd(NO3)_2_ based on the morphological, quantitative characters and yield potential, and were propagated next year (2018–2019) to check the stability of yield traits (Fig 1). In M_3_ generation, eighteen mutant lines viz., mutant A (isolated at 0.10% caffeine), mutant B (isolated at 0.25% caffeine), mutant C (isolated at 0.50% caffeine), mutant D (isolated at 0.75% caffeine), mutant E (isolated at 1.0% caffeine), mutant G (isolated at 0.10% EMS), mutant H (isolated at 0.25% EMS), mutant I (isolated at 0.50% EMS), mutant J_1_ (isolated at 0.75% EMS), mutant J_2_ (isolated at 1.0% EMS), mutant L (isolated at 20 ppm Pb(NO_3_)_2_), mutant M (isolated at 40 ppm Pb(NO_3_)_2_),mutant N_1_ (isolated at 60 ppm Pb(NO_3_)_2_), mutant N_2_ (isolated at 80 ppm Pb(NO_3_)_2_), mutant P (isolated at 20 ppm Cd(NO_3_)_2_), mutant Q (isolated at 60 ppm Cd(NO_3_)_2_), mutant R (isolated at 80 ppm Cd(NO_3_)_2_), mutant S (isolated at 100 ppm Cd(NO_3_)_2_) (Table 1) were screened based on morpho-physiological, biochemical traits. Further, molecular analysis and profiling of mutant lines were performed for analyzing genotypic divergence among all the mutant genotypes along with the control. Least significant difference (LSD) of selected high yielding mutants was evaluated to check significant differences at 5% and 1% (P *<*0.05, 0.01), between mutant and control genotypes for protein and mineral content as well as the morphology of stomatal traits. A correlation coefficient matrix was calculated between yield and biochemical parameters (protein and mineral elements) in high yielding mutants in M_3_ generations. The value of variables of one trait is correlated with the values of the variable of other traits. Data were compiled using a correlation matrix, which was also used as a diagnostic tool for more sophisticated analysis.

### Seed protein and mineral element

The estimation of seed protein in high-yielding mutants seeds were carried out in M_3_ generation by “Lowry Assay: Protein by Folin reaction”. Mineral elements like Iron (Fe), zinc (Zn), and copper (Cu) were determined in the seeds of high-yielding mutants using atomic absorption spectrometry (AAS) technique proposed by Gupta [8]. The significance level of the data was evaluated using Duncan’s multiple range test.

### Seed protein extraction, quantification, and SDS-PAGE analysis

The seed protein of mutants was distilled out using Jarpa-Parra’s protocol for protein extraction [9]. Seeds were powdered using mortar and pestle. Mortar and pestle were used to grind the seeds. The samples were centrifuged at 8500 rpm for 15 minutes at 23 oC after being incubated at room temperature for one hour on a rocker with 1g of flour dissolved in 10 ml of DW at pH 9. The supernatant was accumulated, and its pH was gradually brought down to 4.2 with 0.1 mol/1HCl, and retained it for protein precipitation overnight at 4°C. The sample mixture was then centrifuged for 30 minutes at 1590 rpm, and the dry pellet was kept at 4°C for further research. Protein amount was estimated in M3 generation by using “Lowry Assay: Protein by Folin reaction [10] methodology using bovine serum albumin (BSA). The protein extracts were characterized using SDS-PAGE analysis in accordance with Laemmli’s [11] and He’s [12] detailed explanations of the SDS-PAGE methodology. Coomassie Brilliant Blue staining solution was applied to the gel overnight in order to make it visible.

### Isolation, purification, quantification, and RAPD analysis of genomic DNA

Young leaves were collected from selected mutants and control plants for DNA extraction. DNA of mutants and control plants was extracted using CTAB (Cetyl trimethyl ammonium bromide) method with slight modification as suggested by Agbagwa *et al*. [13] for legumes. DNA samples were quantified and purified generally by spectrophotometric measurements or agarose gel electrophoresis. Extracted genomic DNAs were used to perform PCR based amplification of RAPD fragments followed by RAPD analysis using decamer arbitrary primers. The PCR amplification reaction mixture 20 μl consisted of 25ng genomic DNA template, 1x PCR buffer (50 mM KCl, 10mM Tris HCl, pH 9.0 and 1.5 mM MgCl_2_), 0.2 mM of each dNTPs, 0.5 μM of RAPD primers and 0.3 U Taq DNA polymerase. In PCR tubes, a total of 10 ng of genomic DNA were added altogether as a template. An initial denaturation at 94°C for 3 minutes was followed by 35 cycles of denaturation for 45 seconds, annealing for 1 minute at 36°C, elongation for 2 minutes at 72°C, and final extension for 7 minutes at 72°C in PCR amplification. The PCR products were separated by electrophoresis at 100 volts for three hours on a 1.5% agarose gel in 1xTAE buffer, and then observed with EtBr staining to make them visible.

#### Data scoring and divergence analysis

The reproducible RAPD bands were recorded and scored as either presence (1) or absence (0) for each mutant of lentil, and binary qualitative data was constructed. The binary data was used to generate Jaccard’s similarity coefficient for RAPD markers. Jaccard’s similarity coefficient was employed to determine the pair-wise genetic similarities using the NTSYS-pc-2.02 software. A dendrogram was constructed using the unweighted paired group method with an arithmetical average (UPGMA).

Based on the detected polymorphic and monomorphic bands, the polymorphism percentage (%) was estimated. The following formula was used to get the polymorphism percentage:

$$Percentage\;of\;polymorphism=\frac{Number\;of\;polymorphic\;bands}{Total\;number\;of\;bands}\times 100$$

### Physiological studies

The stomatal analysis of high-yielding mutants was performed using SEM [SEM(JEOL), JSM-6510LV, JAPAN]. The leaf samples were collected and fixed for four hours at 2.5% glutaraldehyde in 0.05M phosphate buffer (pH = 7.1), and then dehydrated in an ethanol series. Samples were placed in pure isoamyl acetate for an hour after being dehydrated. Additionally, samples were dehydrated using a liquid CO_2_ Carl Zeiss EVO SEM critical point dryer before being coated in gold-palladium. The samples were evaluated at an accelerating voltage of 10 kV with x500 and x3000 magnifications.

## Result

### Genotypic variability among induced mutants of *Lens culinaris* using RAPD markers

Genetic variability in lentil mutants was confirmed using RAPD markers (Table 1). In the present study, 10 RAPD primers (OPA-02, OPA-05, OPA-07, OPA-09, OPB-03, OPA-04, OPC-05, OPD-02, OPK-10, OPL-14) were tested for molecular characterization and genetic diversity of M_3_ mutants based on their ability to detect particular and polymorphic amplified products within the mutants.

**Fig 2:**
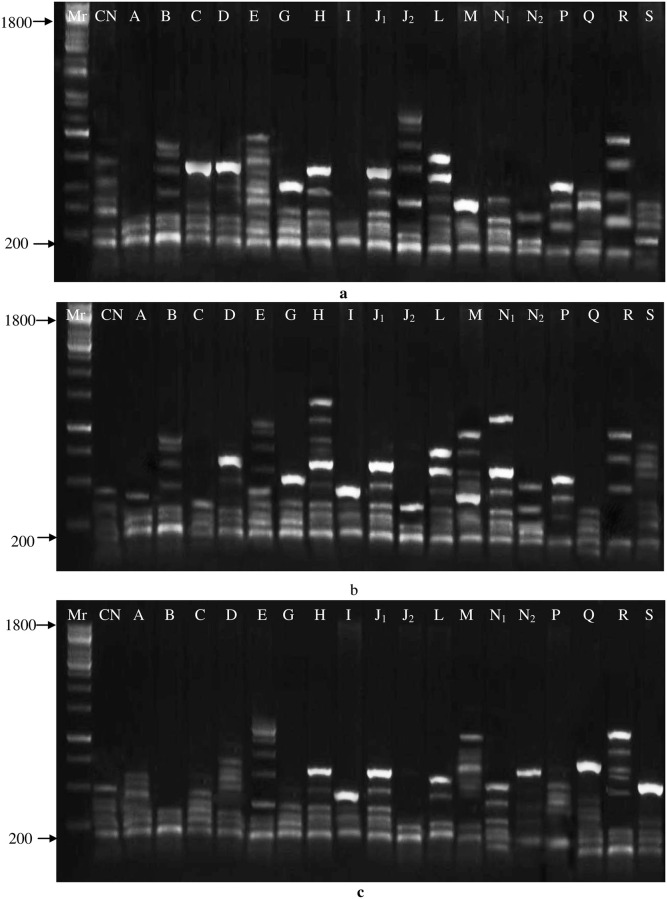
DNA band profile of eighteen different mutants of *Lens culinaris* and control plant using RAPD primers OPA-05, OPB-03 and OPK-10. *(Mr = marker, CN = control).

The heterozygosity of RAPD markers ranged from 50.00–90.90% with an average of 71.04%. In primer OPA-05, the polymorphism percentage was highest (90.90%), followed by OPB-03 (77.27%) and OPK-10 (81.81%), while the lowest polymorphism (50.00%) was recorded in primer OPL-14. (Table 2). Gel images of primers (OPA-05, OPB-03, OPK-10) showing polymorphic and monomorphic bands are presented in (Fig 2a–2c). The total polymorphic percentage of 10 primers was observed at 710.44%. On average, polymorphic bands per primer were 6.7 and monomorphic bands per primer were 2.6, while each primer showed 9.3 average bands. The average polymorphism percentage per primer was recorded to be 71.04% (Table 2).

**Table 2:** Polymorphism information and amplification patterns of 10 RAPD primers during RAPD analysis in induced mutants of *Lens culinaris* Medik.

S. No.	Primers	Nucleotide sequence (5ˈ-3ˈ)	No. of polymorphic bands	No. of monomorphic bands	Total no. of bands	Polymorphism (%)	G+C content (%)	Range (bp)
1	OPA-02	TGCCGAGCTG	6	3	9	66.66	70	200–1200
2	OPA-05	AGGGGTCTTG	10	1	11	90.90	60	200–1600
3	OPA-07	GAAACGGGTG	8	3	11	72.72	60	400–1200
4	OPA-09	GGGTAACGCC	5	2	7	71.42	70	600–1500
5	OPB-03	CATCCCCFTG	7	2	9	77.77	60	200–1600
6	OPB-04	GGACTGGAGT	6	3	9	66.66	60	200–1800
7	OPD-02	GGACCCAACC	5	3	8	62.50	70	200–1200
8	OPC-05	GATGACCGCC	7	3	10	70.00	70	400–1800
9	OPL-14	GTGACAGGCT	4	4	8	50.00	60	400–1600
10	OPK-10	GTGCAACGTG	9	2	11	81.81	60	200–1200
		TOTAL	67	26	93	710.44	-	200–1800
		Avg./primer	6.7	2.6	9.3	71.04		

**Fig 3:**
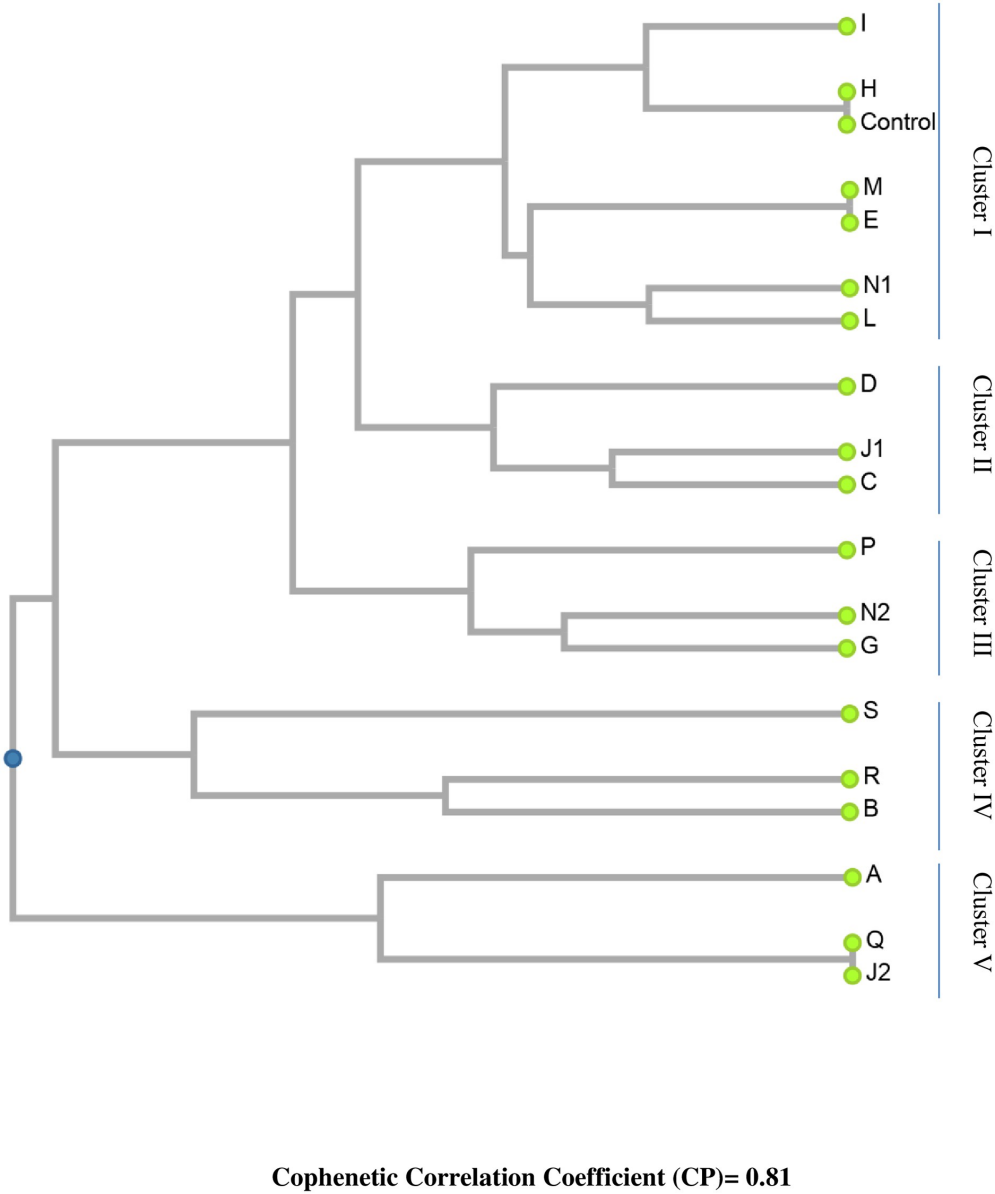
Genetic relationship among control plant (CN) and 18 induced mutants as revealed by the amplification patterns of RAPD primers using Jaccard’s similarity indices and UPGMA method of clustering.

A similarity matrix was produced by comparing the RAPD profile pair-wise using both shared and unique amplification products. The range of the Jaccard-based similarity coefficient was 0.22–1.00. The maximum genetic similarity coefficient (1.00) was observed between control and mutant H; mutant M and E; mutant Q and J_2_, whereas the least significant coefficient (0.22) was observed between mutant N_2_ and B and between mutant R and J_1_ (Table 3). A dendrogram was created owing to the unweighted pair group method using arithmetic average (UPGMA) representing the most probable genetic relationship among the mutants and control (Fig 3). It was observed that all 18 selected *Lens culinaris* mutants along with control plant were grouped into five major clusters. Cluster I is comprised of six mutants (I, H, M, E, N_1,_ and L) along with control (CN). Cluster II combined three mutants (D, J_1,_ and C) obtained in different concentrations of caffeine and EMS. Cluster III grouped three mutants (P, N_2,_ and G) obtained in several mutagenic EMS, Pb(NO_3_)_2,_ and Cd(NO_3_)_2_ concentrations. Cluster IV comprises mutants S, R and B, whereas cluster V comprises mutants A, Q, and J_2_. In cluster I, mutant H closely resembled control and mutant M resembled mutant E, while in cluster V, mutant Q closely resembled mutant J_2_ with the highest similarity index (1.00). The most distantly related mutants were N_2_ and B and mutant R and J_1_ showing lowest similarity coefficient (0.22) (Fig 3). According to the mutants’ estimated cophenetic correlation coefficient (CP), which was 0.81, the dendrogram’s pair-wise distances between the original, unmodeled data points were rather reliably preserved.

**Table 3:** Genetic similarity matrix among control plant (CN) and induced mutants as revealed by the amplification patterns of RAPD primers using Jaccard’s similarity indices.

	CN	A	B	C	D	E	G	H	I	J_1_	J_2_	L	M	N_1_	N_2_	P	Q	R	S
CN	1.00																		
A	0.42	1.00																	
B	0.55	0.42	1.00																
C	0.71	0.60	0.50	1.00															
D	0.71	0.60	0.50	0.66	1.00														
E	0.71	0.60	0.50	0.66	0.42	1.00													
G	0.71	0.60	0.50	0.66	0.66	0.66	1.00												
H	1.00	0.42	0.55	0.71	0.71	0.71	0.71	1.00											
I	0.85	0.50	0.44	0.57	0.57	0.83	0.83	0.85	1.00										
J_1_	0.85	0.50	0.44	0.83	0.83	0.57	0.83	0.85	0.71	1.00									
J_2_	0.28	0.66	0.28	0.40	0.40	0.40	0.40	0.28	0.33	0.33	1.00								
L	0.75	0.42	0.75	0.71	0.50	0.71	0.50	0.75	0.62	0.62	0.28	1.00							
M	0.71	0.60	0.50	0.66	0.42	1.00	0.66	0.71	0.83	0.57	0.40	0.71	1.00						
N_1_	0.86	0.50	0.62	0.83	0.57	0.83	0.57	0.85	0.71	0.71	0.33	0.85	0.83	1.00					
N_2_	0.57	0.40	0.22	0.50	0.50	0.50	0.80	0.57	0.66	0.66	0.50	0.37	0.50	0.42	1.00				
P	0.71	0.33	0.33	0.42	0.42	0.66	0.66	0.71	0.83	0.57	0.40	0.50	0.66	0.57	0.80	1.00			
Q	0.28	0.66	0.28	0.40	0.40	0.40	0.40	0.28	0.33	0.33	1.00	0.28	0.40	0.33	0.50	0.40	1.00		
R	0.33	0.33	0.71	0.25	0.25	0.42	0.25	0.33	0.37	0.22	0.40	0.50	0.42	0.37	0.28	0.42	0.40	1.00	
S	0.57	0.40	0.57	0.50	0.50	0.50	0.28	0.57	0.42	0.42	0.50	0.57	0.50	0.66	0.33	0.50	0.50	0.50	1.00

CN = Control and A to S = mutant’s code

### High yielding mutant lines’ seed protein content and SDS PAGE analysis

The amount of seed protein of lentil mutants is given in Table 4. A substantial improvement on an average seed protein content in the mutants C and I, and a minor protein content enhancement in the mutant A, G, H, J_1,_ and M were recorded when compared to control. The mutant C showed the greatest increase in seed protein content (26.21%), followed by the mutants I (25.50%), A (25.29%), M (25.24%), J1 (24.82%), G (24.64%), and H (24.55%). whereas a slight decrease in seed protein content was found in mutant P (24.32%) concerning control (24.36%).

**Table 4:** Isolated mutants with high yield and protein content in M_3_ generation.

Mutant code	Mutagens	Concentrations (%/ppm)	Yield (g)	Protein content (%)	Remarks
Control	**-**	-	2.35	24.36	Normal yield
A	Caffeine	0.10%	3.72	25.29	Increased yield
C	Caffeine	0.50%	3.94	26.21**	High yield
G	EMS	0.10%	4.43	24.64	High yield
H	EMS	0.25%	3.46	24.55	Increased yield
I	EMS	0.50%	3.03	25.50*	Moderately increased yield
J_1_	EMS	0.75%	3.58	24.82	Increased yield
M	Pb(NO_3_)_2_	40ppm	2.88	25.24	Moderately increased yield
P	Cd(NO_3_)_2_	20ppm	2.79	24.32	Moderately increased yield

Values with an asterisk (*) and (**) differ significantly at the 5% and 1% level (p< 0.05 and p< 0.01) respectively.

**Fig 4:**
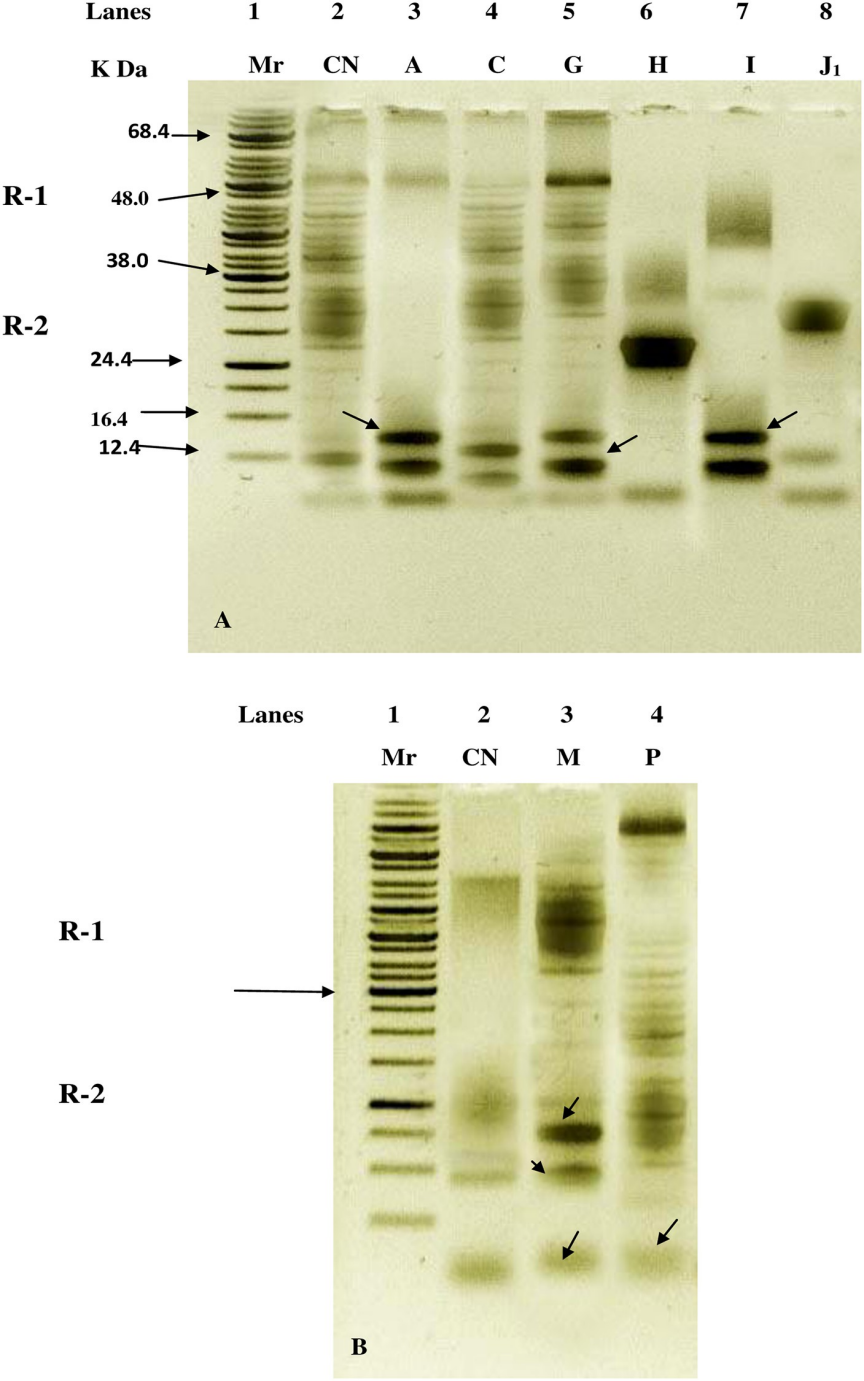
SDS-PAGE protein profile of control and high yielding mutants induced by Caffeine, EMS, Pb(NO_3_)_2_ and Cd(NO_3_)_2_ in M_3_ generation.

The electrophoretic analysis using SDS-PAGE was performed to determine genetic variability, and the variation in seed storage protein pattern in high-yielding mutants. The SDS-PAGE profile of proteins is presented in (Fig 4A and 4B). All of the seed protein extracts had several polypeptides that could be resolved. Based on the banding pattern and molecular weight, the gels were divided into two regions (Region-I and Region-II) for the mutants and control. Region I (R_1_) is comprised of the high molecular weight polypeptide (>38 kDa), and region II (R_2_) is the polypeptide of low molecular weight (<38 kDa). Protein polypeptides in mutants exhibited polymorphic patterns, primarily in region-II (Fig 4A and 4B). Among the mutants, both regions R1 and R2 displayed polymorphism in the banding patterns. In R_1_region, the banding pattern more or less varied in mutants and control. In mutants 1–9 bands were recorded where as in control 9 bands was seen (ranging between 38–68.4 kDa). Mutant bands varied between 2–11 in R2 region, while control bands ranged from 6–11 (ranging between 12.4–24.4 kDa). The most protein bands were present in the mutants C, G, and P, followed by mutants M, A, and I. In general, the occurrence of the banding pattern (ranging between 12.4–38 kDa) was different in mutants compared to control.

### Mineral content in high-yielding mutant lines

**Fig 5:**
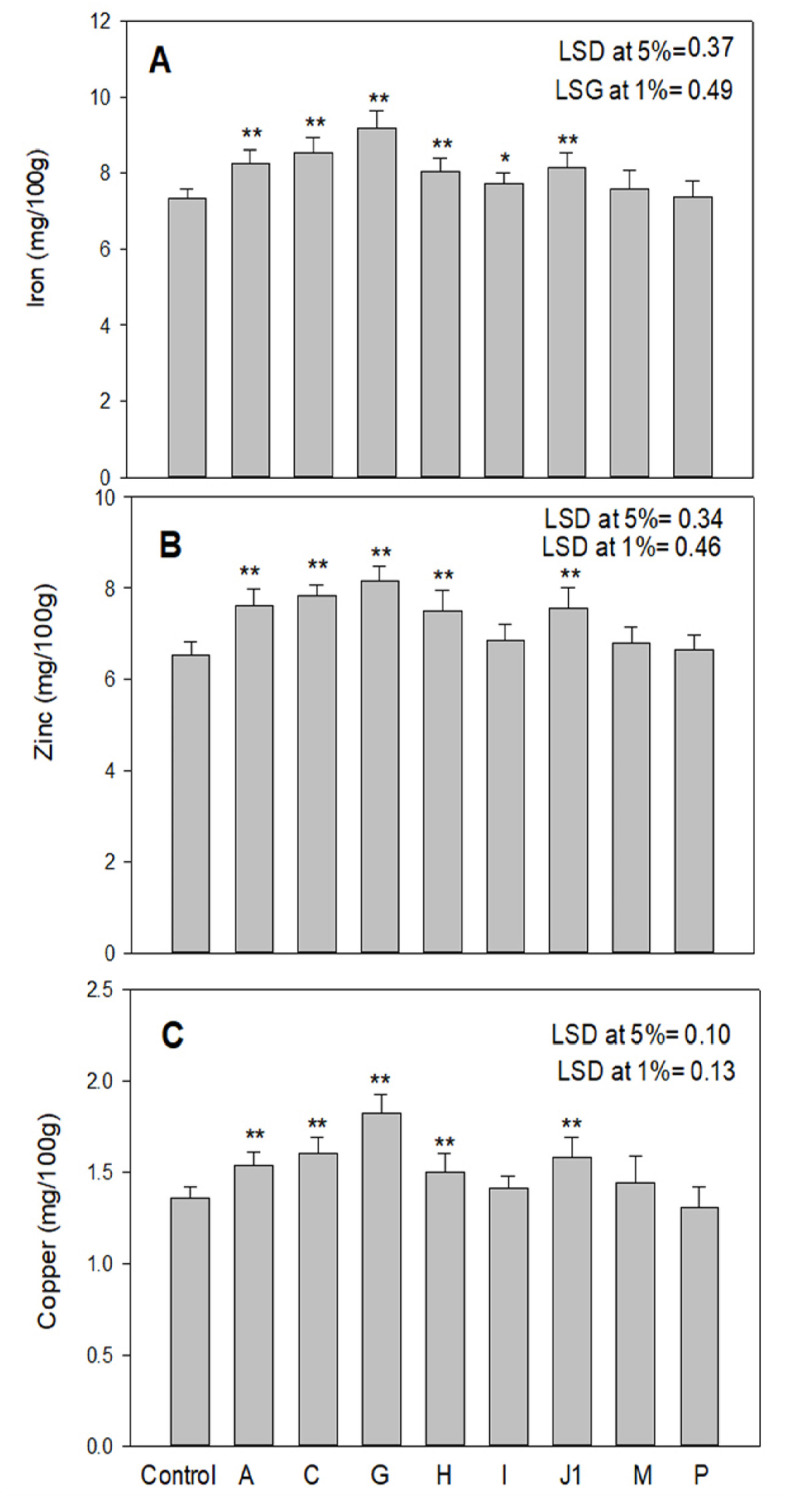
Illustration of iron, zinc and copper content (mg100g^-1)^ in high yielding mutants in M_3_ generation. Results are expressed as mean, ± SE Values with an asterisk (*) and (**) differ significantly at the 5% and 1% level (p< 0.05 and p< 0.01) respectively).

High-yielding mutant lines’ average values and coefficients of variation for various mineral elements, such as Fe, Zn, and Cu, are illustrated in (Fig 5), and it exhibited substantial progressive deviation from control. The greatest improvement in Fe and Zn content (9.19 mg 100^-1^g and 8.16 mg 100^-1^g) was observed in the mutant G. The increase was significant at the 1% level. Mutant G was found to have the highest Cu content (1.82 mg 100-1g) significantly at 1%, while mutant P had a slightly lower Cu content (1.31 mg 100–1) as compared to control. (1.36 mg 100^-1^g).

### Character association studies

Table 5 presents the information on the correlation coefficient between various pairs of yield, protein, and mineral content (Fe, Zn, and Cu) in high-yielding mutants. With the exception of mutant P, the protein content and seed yield of high-yielding mutants showed a substantial positive correlation coefficient. The high-yielding mutant I dominated the character association analysis by showing maximum positive correlation with protein and mineral elements. Correlation trends between mineral elements and yield and among mineral elements themselves in high-yielding mutants have been changed significantly in both positive and negative ways (Table 5).

**Table 5:** Correlation coefficient matrix for seed protein and mineral elements content in high yielding mutants in M_3_ generation.

**Mutant code**	**Characters**	**Yield**			
**Control**		**+.459**			
A		+.618			
C		+.424			
G		+.406			
H		+.757*			
I	**Protein**	+.764*			
J_1_		+.650			
M		+.811**			
P		-.340			
		**Yield**	**Protein**		
**Control**		**+.632**	**+.471**		
A		+.519	+.230		
C		+.841*	+ .633*		
G		+.510	+.453		
H	**Iron**	+659*	+.749*		
I	**(Fe)**	+.517	+.842**		
J_1_		+.456	+.453		
M		+.713*	+.647*		
P		+.529	-.373		
		**Yield**	**Protein**	**Fe**	
**Control**		**+.838** **	**+.415**	**+.679** *	
A		+.287	+.657*	+.229	
C		+.373	+.238	+.333	
G		+.409	+.347	+.372	
H	**Zinc**	+.301	+.573	+.452	
I	**(Zn)**	+.416	+.659*	+.686*	
J_1_		+.365	+.448	+.351	
M		+.654*	+.464	+.698*	
P		+.521	-.500	+.730*	
		**Yield**	**Protein**	**Fe**	**Zn**
**Control**		**+.524**	**+.419**	**+.540**	**+.580**
A		+.723*	+.682*	+.903**	+.734*
C		+.612	+.528	+.751*	+.769**
G		+.456	+.374	+.591	+.732*
H	**Copper**	+.603	+.340	+.195	+.740*
I	**(Cu)**	+.680*	+.762*	+.775**	+.382
J_1_		+.391	+.412	+710*	+.447
M		+.275	+.467	+.488	+.677*
P		-.302	+.709	-.370	-.179

*Correlation is significant at 0.05 level (two-tailed)

**Correlation is significant at 0.01 level (two-tailed)

It was observed that the correlation analysis between pairs of characters, such as protein and yield, mineral and yield, mineral and protein, and among mineral elements exhibited mixed effects (positive and negative) of mutagenic treatments in altering the character association strength (Table 5).

### Variation in stomatal morphology in high yielding mutant lines

**Fig 6:**
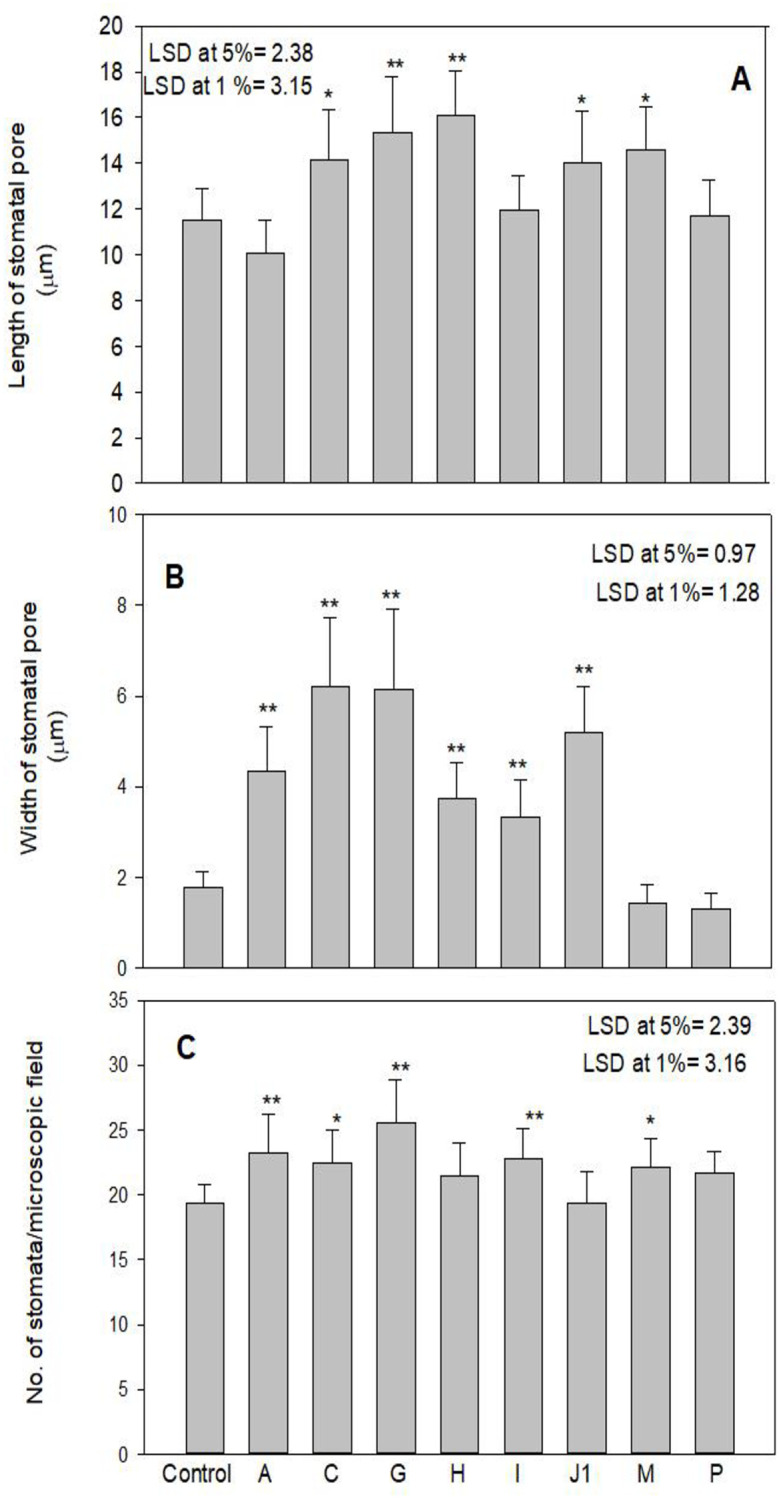
Length, width of stomatal pore and number of stomata per microscopic field in high yielding mutants in M_3_ generation. Results are expressed as Mean ±SE. Values with an asterisk (*) and (**) differ significantly at the 5% and 1% level (p< 0.05 and p< 0.01) respectively).

**Fig 7:**
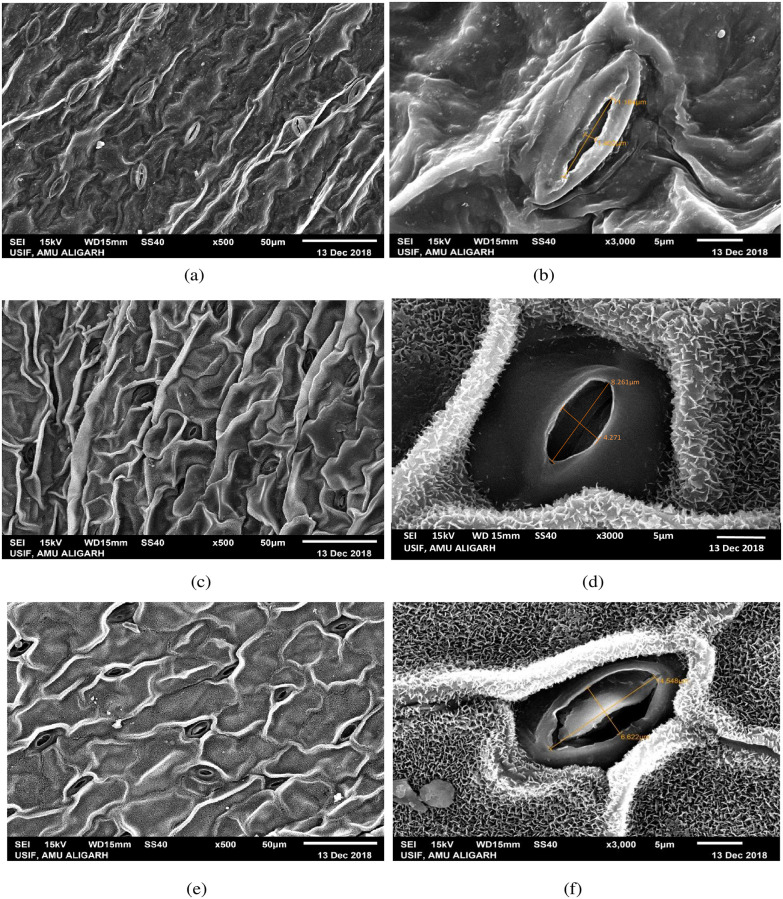
SEM-stomatal photomicrographs of control (a, b), mutant A (c, d) and mutant C (e, f).

**Fig 8:**
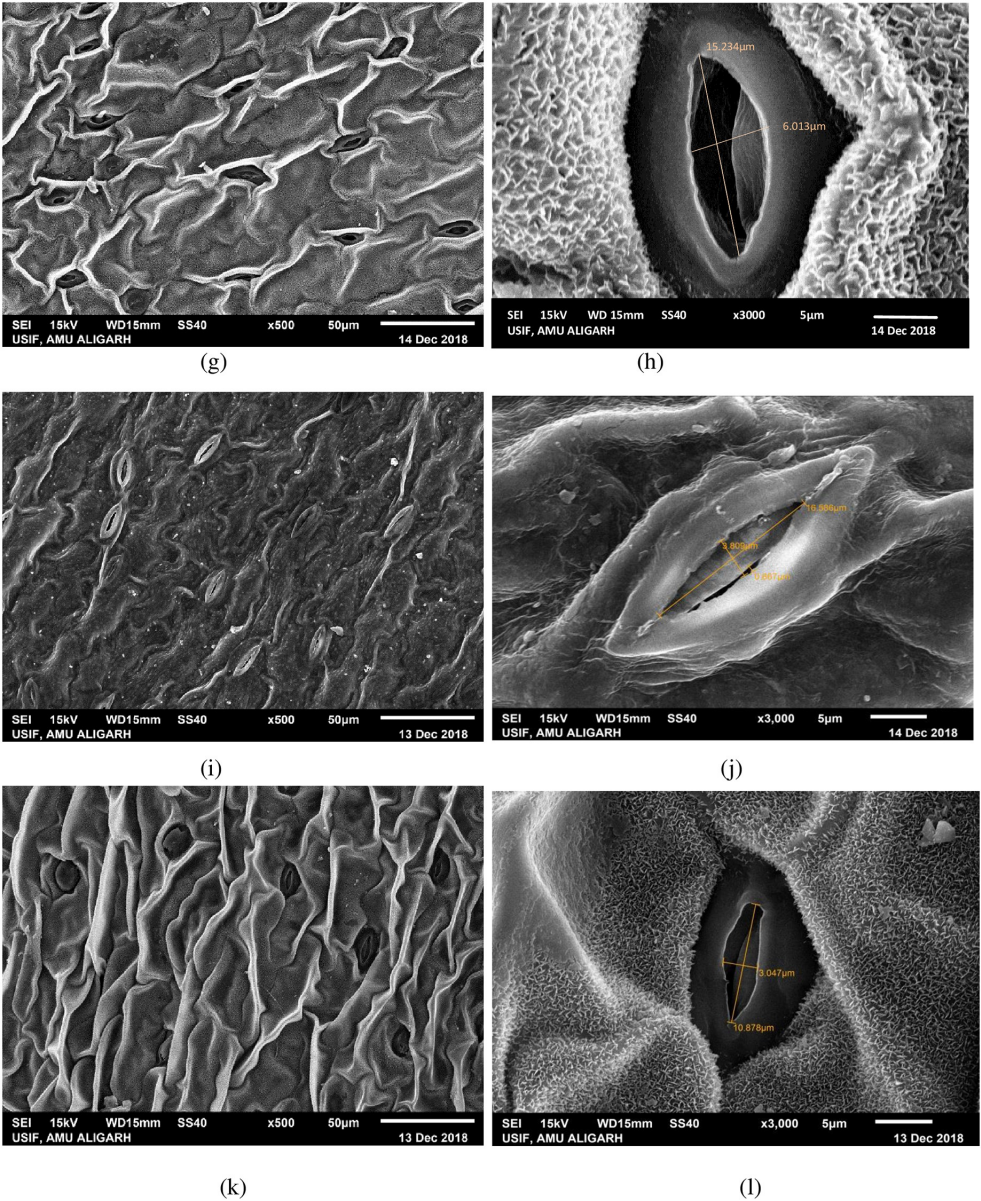
SEM-stomatal photomicrographs of mutant G (g, h), mutant H(i, j) and mutant I(k, l).

**Fig 9:**
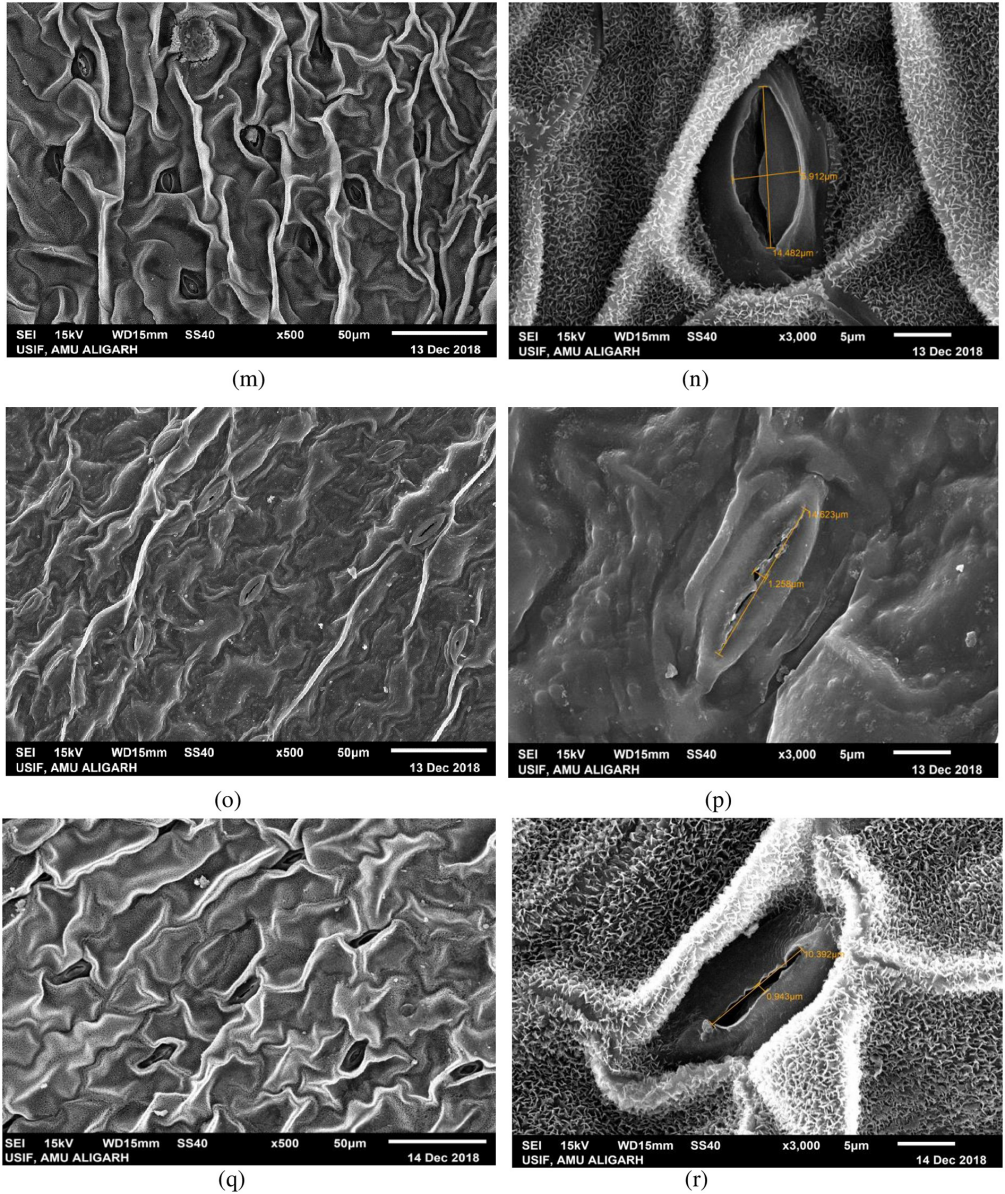
SEM-stomatal photomicrographs of mutant J_1_ (m, n), mutant M (o, p) and mutant P (q, r).

Morphological variation in leaf stomata was observed in the form of length and width of stomatal pore and number of stomata per microscopic field in high yielding mutants (Fig 6A–6C). Structural variation in leaf stomata and guard cell features was observed using SEM at x500 and x3000 magnifications and photomicrographed (Figs 7–9). A significant increase in length of stomata was observed in mutants C, G, H, and J_1_, while other mutants showed minor improvement compared to their control. The maximum significant increase in stomatal length was observed in the mutant H (16.08 μm) over control (11.53 μm) (Fig 6A). Mean values for the width of stomatal pore and number of stomata in mutants exhibited considerable positive deviation from control. Mutant C exhibited the highest significant increase in stomatal width (6.20 m), followed by mutant G, and mutant P exhibited the lowest (1.29 m) compared to control (Fig 6B). The increase was significant at a 1% level in mutants A, C, G, H, I, and J_1_. The number of stomata per microscopic field was found to be maximum (25.57) and significant at a 1% level in mutant G, whereas mutant J_1_ showed the lowest number of stomata (19.32) than control (19.40) (Fig 6C). Stomatal pore was usually open in the control plant (Fig 7a and 7b), whereas it was completely open in mutant C, followed by J_1_, A, and G as compared to control. The guard cells of these mutants were turgid (Figs 7c–7f and 8g, 8h, 8m and 8n), while the guard cells of mutant H and I differed in shape and stomatal pore of guard cells, and moderately open as compared to control (Fig 8I–8l). In mutant M and P, the stomatal aperture was slightly closed, and guard cells became flaccid and varied in shape compared to control (Fig 9o–9r).

Additionally, the investigation revealed that high yielding mutants’ guard cells varied in size and shape. Guard cells of the mutants A, C, and G were oval in shape, and their stomatal pores were fully open (Figs 7d, 7f, 8h and 9n), whereas mutant H showed boat shape guard cells with a relatively open pore (Fig 8j) compared to control having elongated guard cells. Elongated guard cells with irregular margins were observed in mutants I and P (Figs 8l and 9r), while elongated guard cells with dentate margins were found in mutant M (Fig 9p).

## Discussion

### Genotypic variability by RAPD techniques

The best method for examining mutagenic-induced genetic variability is DNA markers. Additionally, the interaction of mutagens with DNA results in changes to gene expression and unique DNA profiles [14]. OPA-05 primer yielded the highest level of polymorphism in the current investigation (90.90%). Sheikh et al. [15]’s report of a 90.6% polymorphism ratio in five lentil accessions using RAPD primer supports this finding. The current findings also suggested that various mutants caused by mutagens exhibit genetic diversity. In comparison to RAPD investigated by [16–18], which had values of 54%, 62.7%, 60.37%, and 54% the PCR amplification using 10 RAPD primers produced reproducible bands with average polymorphic ratio of 71.04%.

The significant RAPD profile changes in the current study were the loss of normal bands and the emergence of novel bands with different intensities in the mutants comparing to the controls. A point mutation, DNA damage and complex chromosomal rearrangements caused by caffeine, EMS, Pb(NO3)2, and Cd(NO3)2 may be responsible for the loss of normal bands. According to Girija et al. [19], following mutagenesis treatments that can decrease the number of taq DNA polymerase binding sites, the disappearance of some bands and variations in band intensities might be correlated to the amount of photoproduct in the DNA template. Emergence of new bands detected by RAPD can be attributed to various DNA structural modifications such as breaks, insertion, deletions and transpositions. According to Bhat et al. [20], genotypic diversity in mutant plants may occur from new bands appearing due to mutation and bands disappearing due to DNA damage., Atak et al. [21] observed significant variation using RAPD markers across mutants caused by varied dosages of atrazine and radiation in soybean. Aslam et al. [22] also reported polymorphism in the flower mutant of Capsicum annuum due to an altered DNA banding pattern. The RAPD markers rapidly perceive a great percentage of polymorphism leading to the generation of genetic maps, and it was recorded by several researchers in various crops like Helianthus annuus [23] and Hordeum vulgare [24]. The detection of percentage verification, hybrid identification, cultivar characterization, and purity testing in a variety of crops have all been accomplished effectively using the RAPD approach.

### Proteomic studies in high yielding mutant lines

In the present investigation, high yielding mutants were evaluated in order to evaluate potential improvements in seed yield and protein content. Some researchers have faced problems in combining the two aspects, i.e., high yield with high protein content [25], while others reported their possibility [26, 27]. In certain high yielding mutants, such as mutant C and I, the total seed protein content was found to have significantly increased concerning control. Similar results were noticed by [28, 29]. Moreover, a few mutants, including mutants A, G, H, and J1, showed a insignificant improve in protein content as reported earlier by [30] in black gram. A slight reduction in protein content was also reported earlier by Ignacimuthu and Babu [31].

In the current work, high yielding *Lens culinaris* mutants were subjected to SDS-PAGE examination of seed storage protein, which revealed polymorphic alterations in peptide chains as variations in the number and intensity of bands. The current analysis found both divergence from the control and among mutant lines. The former research of the several scientists [32, 33] produced comparable outcomes. With comparison to the corresponding control and other mutants, the mutant M displayed a higher number of distinct bands and significant banding pattern variance. Induced diversity in expressed polypeptides among isolated mutants of lentil was evident in the gel picture to a significant extent. The distinctive band of seed storage protein profiles, which may also be employed as markers for the sighting of novel bands in mutants of chickpea [34], *Vicia faba* [35], and cowpea [36], was mentioned by many researchers as a tool for evaluating genotypic variation brought on by mutagens. Gene mutations would be visible in the polypeptides produced by the protein synthesis mechanism as well as in their expression profiles [37] because genes command and control this process. According to Hong *et al*. [38], SDS-PAGE analysis aids in screening of stress tolerant mutants with high yield. When proteins from the mutants A, G, I, M, and P were screened molecularly, new protein bands of various molecular weights were discovered. These new bands may be the result of transition mutations in the DNA. The nucleotide sequences of alleles can differ, which can result in the production of multiple proteins or various type of amino acids. These proteins contain the genetic information needed to generate different morpho-physiological and anatomical traits in plants. Mutagenic prompted modifications in the electrophoretic pattern of seed protein have been recorded earlier by several workers in different crops, such as in *Glycine max* [39], *Cicer arietinum* [40], *Vicia faba* [35], *Lens culinaris* [29] and cowpea [36]. Based on the above results, it could be possible to evaluate genetic variability and to distinguish the genetic pattern of protein bands of high yielding mutants through SDS-PAGE analysis

### Mineral content in high yielding mutant lines

A considerable increase in the mineral concentrations of Fe, Zn, and Cu was seen in some mutants, which may solve the problem of mineral nutrient deficiency. The study exhibited that combining high micronutrients with high seed yield is possible. The results reported in the current study are in validation with the work of different workers in various crops such as faba bean [41], chickpea [42] and lentil [29]. There has been a lot of study done on the production of mutations in lentil agro morphological features, but little has been done in-depth on the induction of changes in seed mineral elements like Fe, Zn, and Cu. There are just a few high yielding mutants that have more mineral elements (Fe, Zn, and Cu) in them. Based on the aforementioned information, it has been determined that selection for yield traits may be combined with genetic improvement of the nutritional content of lentils for potential mutant generation.

Observations of protein and mineral elements analysis showed no significant difference between control plants and mutant plants in terms of coefficient of variation in protein and minerals. Thus, further advancement of the seed protein and mineral components cannot be anticipated through selection of such mutants. However, certain mutants showed notable variations in the coefficient of variation for seed protein content and mineral element from the control, suggesting the potential for selection in such mutants for future advancements in the selection process.

### Correlation studies

A number of pairs of polygenic traits in the mutant lines chosen in the M3 generation showed a correlation coefficient that significantly varied in the desired direction. Protein content and yield showed a positive and significant association in several mutants (M, I), suggesting that these mutant lines have a strong potential for simultaneously improving protein and yield. However, certain mutant lines displayed positive correlations that were not statistically significant, indicating that there was little chance for simultaneous improvement in yield and protein in these mutant lines. The research found that, with the exception of mutant P, the association between mineral elements and protein content was always positive. Due to a negative association between these two features, this study shows that simultaneous improvement of protein content and yield simultaneously is not attainable by mutation breeding in mutant P. The shift in the positive and negative correlations between the mineral components and seed yield as well as among the minerals suggests that genetic makeup plays a part in influencing the strength of trait correlations. Similar results have previously been noted by other researchers [25, 33, 36, 43–45]. The selection of high yielding nutritional mutants for subsequent breeding operations benefited from the mutants having promising correlations between yield and mineral elements, both significant and insignificant. Other researches have noted the substantial and positive correlation between the mineral elements and high seed yield [46], minerals and proteins [47], and among the mineral elements. The possible reason for the trait differential correlation matrix might be agonistic and antagonistic metabolic pathways [48].

The findings indicated that genotypic variation in these traits should be taken into consideration for high yielding mutants, as they showed a positive correlation with yield, mineral elements, and protein content. This would hasten the development of more productive and nutrient-dense varieties for farmers. So, mutagen-induced genetic variability could be used for genetic research as well as improving lentil yield and nutritional value.

### Stomatal morphology in high yielding mutant lines

The purpose of the study was to comprehend the changes in stomatal structures brought on by mutagens in numerous mutants of lentil. The increased stomatal size was also reported earlier by [49–52], as observed in the present study. The stomata control gaseous exchange between the leaf and atmosphere, govern CO_2_ uptake for photosynthesis, and determine plant productivity. So, enhance stomatal size (length and width) and the number of stomata in high yielding mutants leading increase degree of gaseous exchange between plant tissues and environment, eventually increasing photosynthetic process and plant yield. Moreover, in some mutants, the length and width of stomatal pores slightly reduced compared to control, but their number increased, i.e., it showed negative correlation between stomatal size and stomatal density in those mutants. This result is supported by [53, 54]. The stomatal pore was completely open, and guard cells were turgid in mutant C, J_1_, A, and G, while the stomatal aperture was slightly closed, and guard cells became flaccid in mutant M and P in the present study. Since yield and proteins were negatively correlated in mutant P, the stomata might have played negatively in CO_2_ fixation; the yield was affected as compared to other high yielding mutants. However, the slight increase in the yield over control might be due to an increase in number of stomata. The opening of the stomatal pore is elevated by photosynthetically sufficient light, low levels of CO2 with high humidity, while stomatal closure is encouraged by the conditions of darkness, low humidity, high temperature, and high CO_2_ concentration [55–57]. Buckley [58] reported that the number of stomata is related to photosynthetic activities. According to Sarwar *et al*. [59] stomatal size and density are directly related to different physiological processes of plants, such as photosynthesis, and increased photosynthetic activity correlates with greater yield. The enhancement/reduction in stomatal size and frequency of high yielding mutants was possibly due to induced genetic damages and mutations. Induced mutagenesis caused alterations in the structure of stomata, size and frequency that might be a way to create superior high yielding mutants to increase productivity for future improvement programs.

## Conclusion

The study concluded that the variations in physio-biochemical and molecular characters were a result of perturbations brought on by mutagens’ activity and their interactions with the environment. The accessibility of genetic variability in species is indispensable for crop improvement. Assessment of stomata, protein, mineral, and morphology exhibited enhanced physiological and morphological processes in the high yielding lentil mutants.

In high yielding lentil mutants, SDS-PAGE examination of the seed storage protein revealed polymorphic alterations in polypeptide chains, as evidenced by differences in the number and intensities of bands in comparison to controls and among themselves. It proved that mutagens could be used to induce variation in seed protein banding patterns. The RAPD markers have generated a considerable number of polymorphic mutants that confirm genetic diversity from the control population. The genetic relationship attained from the clustering pattern utilizing RAPD markers can help breeders differentiate between the dissimilar genotypes from clusters and use them in future breeding programs.

The present study’s genetically divergent mutant lines can be utilized either directly as a parents in hybridization programs or as mutant variety for further development and stabilization of lentil traits.

## Supporting information

S1 Raw image (PDF)
