# Supplementary material for: Physio-biochemical analysis and molecular characterization of induced lentil mutant lines
Source: PLoS One. 2022 Oct 24;17(10):e0274937. doi: 10.1371/journal.pone.0274937 (PMC9591049; doi:10.1371/journal.pone.0274937)

Figure 2a
DNA: Lentil mutant lines
Primer:OPA-05

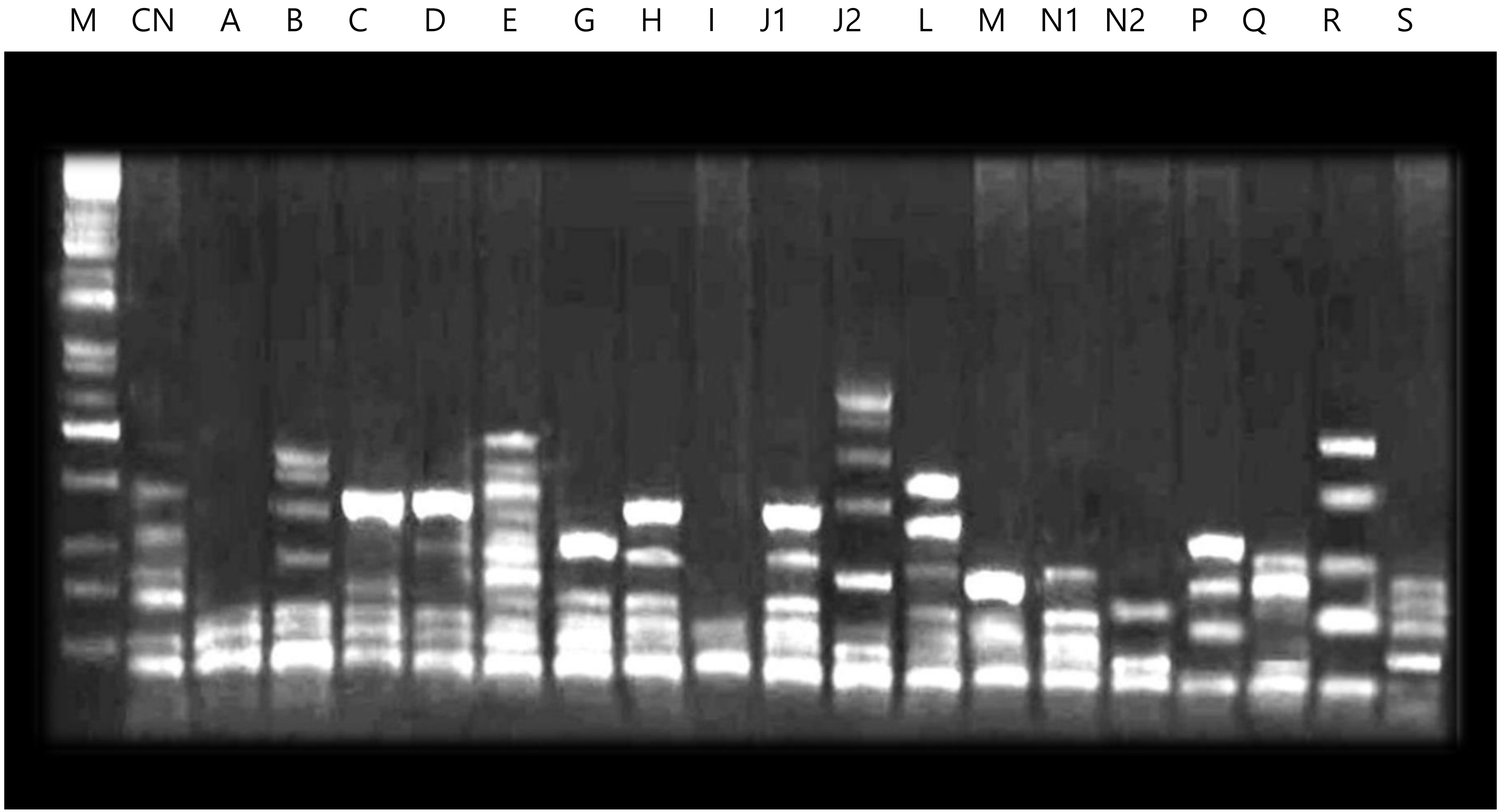

Figure 2b
DNA: Lentil mutant lines
Primer: OPB-03

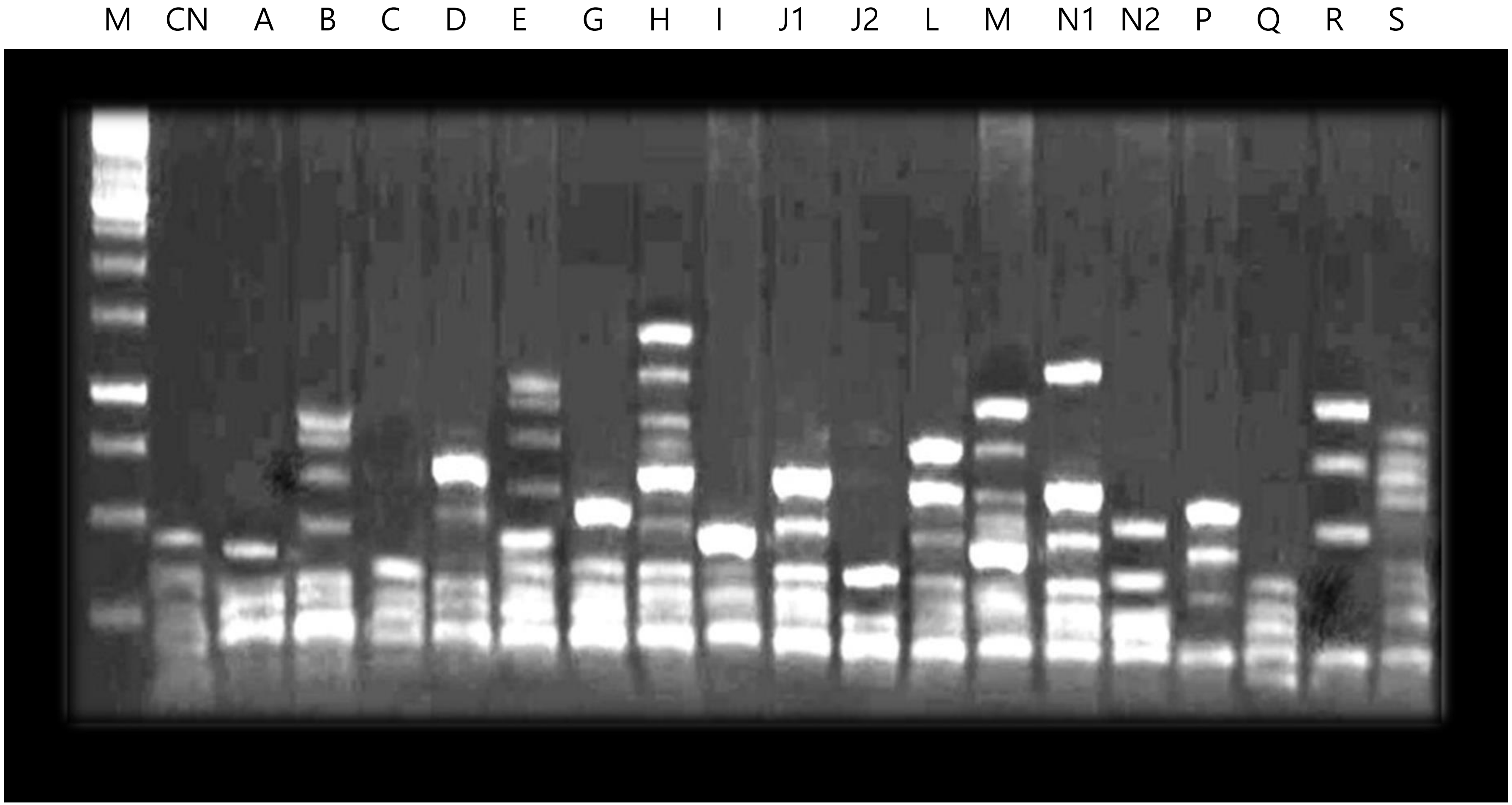

Figure 2c
DNA: Lentil mutant lines
Primer: OPK-10

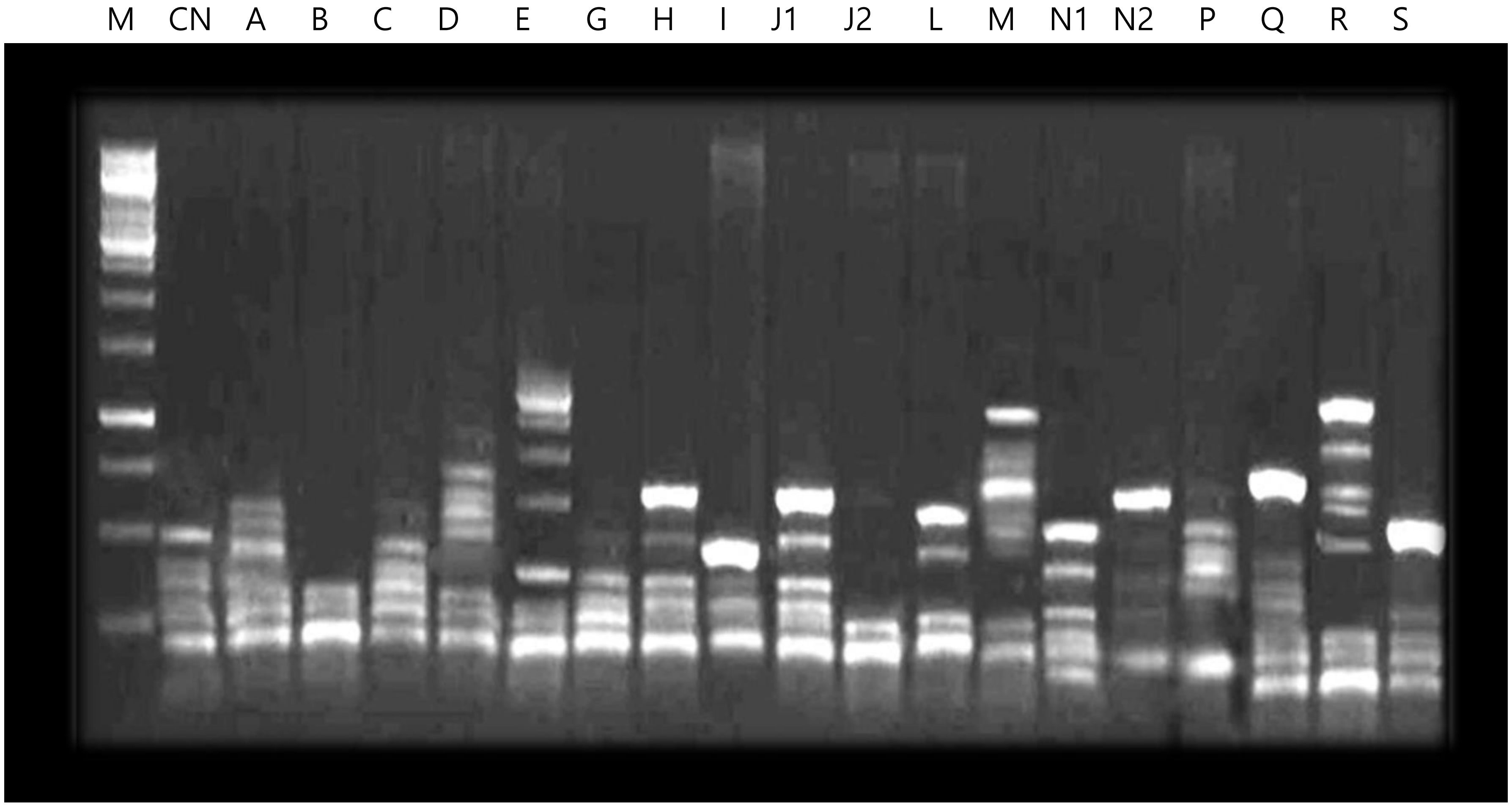

Supplement: S1 Raw image — (PDF) [file pone.0274937.s001.pdf]
